# Clinical Features of 294 Turkish Patients with Chronic Myeloproliferative Neoplasms

**DOI:** 10.4274/tjh.2015.0041

**Published:** 2016-08-19

**Authors:** Neslihan Andıç, Mustafa Ünübol, Eren Yağcı, Olga Meltem Akay, İrfan Yavaşoğlu, Vefki Gürhan Kadıköylü, Ali Zahit Bolaman

**Affiliations:** 1 Osmangazi University Faculty of Medicine, Department of Hematology, Eskişehir, Turkey; 2 Adnan Menderes University Faculty of Medicine, Department of Hematology, Aydın, Turkey; 3 Osmangazi University Faculty of Medicine, Department of Internal Medicine, Eskişehir, Turkey

**Keywords:** Myeloproliferative neoplasms, Survival, Thrombosis, treatment

## Abstract

**Objective:**

Myeloproliferative neoplasms (MPNs) share common clonal stem cells but show significant differences in their clinical courses. The aim of this retrospective study was to evaluate thrombotic and hemorrhagic complications, JAK2 status, gastrointestinal and cardiac changes, treatment modalities, and survival in MPNs in Turkish patients.

**Materials and Methods:**

Medical files of 294 patients [112 essential thrombocythemia (ET), 117 polycythemia vera (PV), 46 primary myelofibrosis, and 19 unclassified MPN cases] from 2 different universities in Turkey were examined.

**Results:**

Older age, higher leukocyte count at diagnosis, and JAK2 mutation positivity were risk factors for thrombosis. Platelet count over 1000x109/L was a risk factor for hemorrhagic episodes. Hydroxyurea treatment was not related to leukemic transformation. Median follow-up time was 50 months (quartiles: 22.2-81.75) in these patients. Patients with primary myelofibrosis had the shortest survival of 137 months when compared with 179 months for ET and 231 months for PV. Leukemic transformation, thromboembolic events, age over 60 years, and anemia were found to be the factors affecting survival.

**Conclusion:**

Thromboembolic complications are the most important preventable risk factors for morbidity and mortality in MPNs. Drug management in MPNs is done according to hemoglobin and platelet counts. Based on the current study population our results support the idea that leukocytosis and JAK2 positivity are more important risk factors for thrombosis than hemoglobin and platelet values.

## Abstract (tr)

**Amaç:**

Miyeloproliferatif hastalıklar (MPH) ortak klonal bir kök hücreden köken almalarına karşın klinik seyirleri belirgin farklılıklar göstermektedir. Bu retrospektif çalışmanın amacı MPH’lardaki trombotik ve hemorajik komplikasyonların, JAK2 mutasyon durumunun, gastrointestinal ve kardiyak değişikliklerin, tedavi şekillerinin ve yaşam sürelerinin incelenmesidir.

**Gereç ve Yöntemler:**

Türkiye’nin iki farklı üniversite hastanesinden 294 hastanın [112 esansiyel trombositemi (ET), 117 polisitemia vera (PV), 46 primer miyelofibozis, 19 sınıflanamayan MPH] kayıtları incelenmiştir.

**Bulgular:**

İleri yaş, tanıda yüksek lökosit sayısı JAK2 mutasyon pozitifliği tromboz için risk faktörü olarak bulunmuştur. Trombosit sayımının 1000x109/L üzerinde olması kanama komplikasyonları açısından risk faktörüdür. Hidroksiüre tedavisi lösemik dönüşümle ilişkili bulunmamıştır. Bu hastalarda: Medyan takip süresi 50 ay (22,2-81,75 çeyrekler) idi. Primer miyelofibrozisli hastalar ET için 179 ay ve PV için 231 ay olan yaşam süreleri ile karşılaştırıldığında 137 ay ile en kısa yaşam süresine sahip hastalardır. Lösemik transformasyon, tromboembolik olaylar, 60 yaş üstü olmak ve anemi yaşam süresinin etkileyen faktörler olarak bulunmuştur.

**Sonuç:**

Tromboembolik komplikasyonlar MPH’da en önemli önlenebilir mortalite ve morbidite nedenidir. İlaç düzenlemeleri çoğunlukla hemoglobin ve trombosit sayımlarına göre yapılmaktadır. Bu çalışmamızdaki hasta popülasyonundan elde ettiğimiz veriler lökositoz ve JAK2 pozitifliğinin hemoglobin ve trombosit sayımlarından daha önemli risk faktorleri olduğu savını desteklemektedir.

## INTRODUCTION

According to the revised World Health Organization (WHO) classification, BCR-ABL-negative chronic myeloproliferative disorders are now referred to as myeloproliferative neoplasms (MPNs) [1,2]. MPNs share common clonal stem cells and phenotypic differences occur due to different molecules affecting signal transduction. The JAK2V617F mutation is an acquired point mutation causing valine-to-phenylalanine substitution at codon 617 on the JAK2 gene. JAK2V617F will be referred to as JAK2 mutation in the text. JAK2 mutations affecting the JAK-STAT signal transduction pathway are found in 90%-95% of patients with polycythemia vera (PV) [3], 50%-70% of patients with essential thrombocythemia (ET), and 40%-50% of patients with primary myelofibrosis (PMF) [4]. JAK2 mutations cannot be used in distinguishing one MPN from another but are useful in excluding reactive hematocrit and platelet elevations and reactive myelofibrosis. Absence of JAK2 mutations cannot exclude the diagnosis of PV, ET, or PMF. Some clinical criteria and bone marrow findings are required for the diagnosis of JAK2-negative MPN [2].

Prognosis of MPNs is determined by thromboembolic and hemorrhagic complications and progression to myelofibrosis and acute leukemia. The cumulative rate of nonfatal thrombosis in PV is 3.8 events per 100 persons per year, and in ET the rate of fatal and nonfatal thrombotic events ranges from 2% to 4% of patient years. Primary myelofibrosis seems less susceptible for thrombotic events as the cumulative percentage is 2.23% per patient years [5]. Age and previous thrombosis are known risk factors for future thrombosis in MPNs. Leukocytosis and JAK2 mutation are shown to be additional risk factors. Extreme thrombocytosis (count over 1000 or 1500x109/L) was found to be related to hemorrhagic complications but not thrombosis [5].

Other complications like gastrointestinal ulcers and echocardiographic changes are also reported. Their importance in the course of the disease is only partially understood [6,7].

Hydroxyurea and anagrelide are the most commonly used drugs in the treatment of MPN. Hydroxyurea was shown to reduce the incidence of thrombotic events in several studies, but there is some evidence that it may increase the risk of leukemic transformation [8]. Anagrelide is effective in reducing platelet counts in ET and PV patients who are resistant or intolerant to hydroxyurea. Risk increment of leukemia has not been shown for this drug [9].

The aim of this study is to evaluate thrombotic and hemorrhagic complications, JAK2 status, gastrointestinal and cardiac changes, treatment modalities, and survival in MPN cases.

## MATERIALS AND METHODS

The medical files of patients diagnosed with Philadelphia chromosome-negative chronic myeloproliferative disease (CMPD) and MPN between 2003 and 2013 were retrospectively examined. Two centers in Turkey entered the study: Eskişehir Osmangazi University and Adnan Menderes University. Diagnoses were made according to PV Study Group and WHO recommendations. The WHO criteria were revised in 2005 after the discovery of JAK2 mutations. In the revision of WHO criteria made in 2008, ‘CMPD’ was changed to ‘MPN’. Patients with significant fibrosis in the bone marrow but otherwise clinically diagnosed with ET by the primary clinician were placed in the category of unclassified MPN and will be referred to here as MPN(u) patients. We included MPN(u) patients in the statistical analysis done for the whole patient group, like statistics of risk factors for thrombosis. On the other hand, we did not include MPN(u) in one-to-one comparisons with the three MPN groups (ET, PV, and PMF) because we wanted to compare the patients with exact diagnoses.

The study was approved by the local ethics committees of both universities. Patients above the age of 16 at the time of diagnosis were enrolled in the study. All consecutively admitted patients during the mentioned period were taken into consideration. Clinical and laboratory parameters were recorded. JAK2 status and other cytogenetic abnormalities and bone marrow findings were evaluated. Treatment modalities, thrombotic and hemorrhagic complications, and gastrointestinal and cardiac findings were noted.

Proteins C and S were studied by the Siemens BCSX coagulometric method and antithrombin was studied by Siemens BNII nephelometric method. Factor V Leiden and prothrombin gene mutations were studied with a Roche 480 II LightCycler by the real-time polymerase chain reaction (PCR) method. Bone marrow samples were cultured in 24-48 h in standard 10 µg/mL colcemid solution without mitogen in order to perform conventional bone marrow cytogenetics. Twenty metaphases were evaluated. A locus-specific LSI D20S108 (20q12) probe was used for fluorescence in situ hybridization (FISH) analysis and 200 cells (metaphase/interphase) were evaluated. JAK2V617F mutations were studied by real-time PCR method with a Roche 480 II LightCycler using the TIB Molbiol LightMix Kit. Bone marrow aspirates and biopsies were evaluated in the pathology and hematology departments of both universities. Increases in megakaryocytes and grades of reticulin fibrosis were defined according to the WHO classification of tumors [10,11].

### Statistical Analysis

Statistical tests were performed using IBM SPSS 20.0 for Windows and p<0.05 was considered significant. The Shapiro-Wilk test was performed for testing normality. The chi-square test was used to compare categorical variables and the Kruskal-Wallis test was used for continuous variables not normally distributed, followed by Dunn’s post hoc test. Survival was assessed using Kaplan-Meier analysis and the log-rank test was used for univariate comparisons. The effect of prognostic factors on survival was analyzed by Cox proportional hazards regression models.

## RESULTS

A total of 294 patients’ medical files were eligible for the study; 143 (48.6%) patients were female and 151 (51.4%) were male. Median age was 60 years (quartiles: 48-79), with a minimum of 16 and maximum of 84 years. Sex and age were not statistically different between patient groups. The number of patients diagnosed with ET was 112 (38.1%), with PV was 117 (39.8%), and with PMF was 46 (15.6%). Nineteen patients who were diagnosed with and treated for ET by the primary physician were later classified as having MPN(u). These patients had no leukoerythroblastosis in peripheral blood and their median platelet count was 1146x109/L (quartiles: 844-1416). All of them had megakaryocytic proliferation in their bone marrow and had neither dysplasia nor prominent granulocytic and erythroid proliferation. Median follow-up time was 68 months (quartiles: 15-81). Six of them had grade 2-3 and 13 of them had grade 3 reticulin fibrosis in their bone marrow. We could not classify these cases as ET or PMF so we classified them as MPN(u).

The longest follow-up period was 311 months (median: 43 months; quartiles: 15.7-77.2). In 103 patients there were no comorbidities, while 155 patients had hypertension and/or diabetes mellitus, and 53 patients had diseases including chronic obstructive pulmonary disease, liver failure, renal failure, congestive heart disease, and arrhythmias including atrial fibrillation. One patient had lung cancer and another had prostate cancer. Eighty-five patients (28%) were smoking cigarettes. The clinical and laboratory characteristics of patients are summarized in Table 1. Splenomegaly and hepatomegaly were significantly more frequent in PMF than in other MPNs. Hemoglobin was significantly higher in PV than in other groups and significantly lower in PMF than in other groups. Platelet count was higher in ET than in other groups. Lactate dehydrogenase (LDH), potassium, and uric acid values were higher in PMF compared to other MPN subtypes. Median LDH was above normal limits in all study groups. Bone marrow findings at diagnosis are summarized in Table 2. Megakaryocytes were more prominent in ET and reticulin fibrosis was more profound in PMF than in other MPNs, as expected.

It was found that 58.5% (38 patients) of ET patients, 86.2% (50 patients) of PV patients, and 70.6% (12 patients) of PMF patients were positive for JAK2 mutation. Patients with PV were carrying JAK2 mutations significantly more so than patients with ET (p<0.001). There were no statistically significant differences between ET and PMF and PV and PMF regarding JAK2 mutation. Conventional cytogenetics and FISH analysis of the bone marrow revealed that 2 patients with ET had trisomy 8 and 1 patient had 5q-, while 3 patients with PV had 20q-, 1 patient had 13q-, and 1 patient had trisomy 20. One patient with ET who had trisomy 8 had developed acute myeloid leukemia.

Thromboembolic complications were seen in 36% (n=108) of patients, while 41.1% of ET patients (n=46), 35% of PV patients (n=41), and 32% of PMF patients (n=15) had thrombotic events. Six other patients who had thromboembolic events were later reclassified as having MPN(u). Most of the thrombotic events were in cerebral arteries (37 out of 108). Approximately half of these cerebral arterial occlusions were seen in ET patients (16 out of 37). Coronary artery disease was the second most common thrombotic complication (32 out of 108). Ten patients had deep vein thrombosis (9.3%). Twelve patients (11.1%) had thrombosis in an intraabdominal vein. Ten patients had both arterial and venous thrombotic attacks and most of them were ET patients (8 out of 10). Diagnostic groups, sex, treatment modality, and bone marrow findings did not differ between patients with or without thrombosis. Older age, higher leukocyte count at diagnosis, and JAK2 mutation positivity were risk factors for thrombosis after univariate analysis. Data are shown in Table 3.

Hemorrhagic complications were seen in 34.4% (n=101) of patients. Almost all (94%) patients with hemorrhagic complications had mucocutaneous or gastrointestinal tract bleeds. There was no statistical significance between MPN groups regarding the frequency and the source of bleeding. Hemoglobin values were significantly lower in patients with hemorrhagic episodes than patients without hemorrhage (p=0.012). Medians and quartiles were 13.1 g/dL (10.3-17) and 14.6 g/dL (12.7-17.1), respectively. Platelets count over 1000x109/L was a risk factor for hemorrhagic episodes; 30.1% (n=59) of patients with platelet count less than or equal to 1000x109/L had hemorrhagic events, whereas 42.9% (n=42) of patients with platelet count over 1000x109/L had hemorrhagic episodes (p=0.030). Leukocyte counts, fibrinogen levels, and treatment modalities were not statistically different between patients with and without hemorrhage.

Electrocardiogram results were considered as normal in 78.2% (n=230) of cases. Atrial fibrillation was present in 7.4% (n=22) and signs of ischemia in 8.2% (n=24) of patients. Echocardiography was performed in 95 patients. Forty-one (43.2%) of them had cardiac valve abnormalities and 10 (10.5%) had pulmonary hypertension.

Upper gastrointestinal endoscopy was performed in 80 patients. Gastritis and duodenitis were frequent findings (56 patients, 70%). Nine patients (11.3%) had ulcers. Nine patients had esophageal varices. Helicobacter pylori testing was done in 56 patients and 53.6% of them were positive.

Treatment modalities are shown in Table 4. Hydroxyurea was the first choice in ET, PV, and PMF cases. Anagrelide was mainly used in ET. Patients who were receiving anagrelide treatment were significantly younger than the patients receiving hydroxyurea treatment [49.5 years (39.7-63) and 60 years (50-69), respectively, p<0.001]. Interferon alpha was used only in 6.5% of MPN patients. Acetylsalicylic acid was used in approximately 80% of PV and ET cases. Anticoagulant drugs were administered after a thromboembolic event in 34 patients (29.1% of thromboembolic events) and the frequency was not different between MPN groups (p>0.05). Thirty-six patients (12.2%) were treated with phlebotomy alone, and 159 patients (54%) received any kind of cytoreductive therapy along with phlebotomy. Seven patients developed acute myeloid leukemia during follow-up. Three of them had ET, 2 of them had PV, and 2 of them had MPN(u). Patients who developed leukemia were not different from others by means of sex, megakaryocyte number, or grade of reticulin fibrosis in the initial bone marrow. Among 3 patients with excess (>5%) blasts in the initial bone marrow, 1 developed leukemia and the other 2 were diagnosed with PMF. Receiving previous treatment with hydroxyurea was not found to be a risk factor for leukemic transformation. Median follow-up time of patients receiving hydroxyurea treatment was 50 months (quartiles: 22.2-81.75). One patient with ET previously treated with hydroxyurea showed transition to myelofibrosis.

Forty-four patients died during follow-up. Among disease-related deaths, thromboembolic events were the main cause for ET patients and progression of the disease was the main cause for PMF patients.

Factors affecting survival in MPN are shown in Table 5. Overall survival of the PMF patients was shorter than in the other MPN groups (p<0.001). Leukemic transformation shortened the survival significantly at 78 vs. 210 months (p<0.001). Mean overall survival of patients with any thromboembolic event was significantly shorter than that of patients without thromboembolic events. In subgroup analysis, the same effects of thromboembolic events were seen in ET patients but not in PV and PMF patients. Arterial or venous nature of the thrombi did not affect the survival time in MPNs. Hemorrhagic complications did not have any significant effect on survival. Survival time was significantly shorter when the patient’s age was 60 years or older at diagnosis (p=0.001). Patients with hemoglobin levels lower than 13 g/dL lived significantly shorter than those with hemoglobin of 13 g/dL or higher (p=0.001). Leukocyte and platelet counts had no significant effects on survival.

We did not find any significant effect of bleeding events or JAK2 mutation status on survival. Treatment methods including acetylsalicylic acid and anticoagulant drugs were not effective on survival for the overall patient population.

## DISCUSSION

This retrospective study was aimed at evaluating the characteristics of MPNs in Turkish patients. Two centers contributed to the study and 294 patients were enrolled. Median age was 60 years and the female/male ratio was 0.9. These two findings were consistent with the literature [12,13]. Although there is knowledge in the literature that ET is more common in women and PV more common in men, we did not find any difference in sex between MPN groups [3,14]. Splenomegaly and hepatomegaly were common findings in physical examination. They were seen in almost 80% of patients with PMF, more commonly than in the other MPN groups. This finding is consistent with the literature, but our frequencies in PV and ET are higher than those reported in other studies [13,14,15]. Even minimal enlargement in the spleen, like 130 mm in a male patient, was noted as splenomegaly in this study. This could be the reason for higher splenomegaly rates in ET and PV patients compared with the literature. Hemoglobin levels were higher in PV and platelet levels were higher in ET, and they were both lower in PMF, as expected (p<0.001). The increased serum LDH levels in PMF may reflect increased disease bulk as well as the proliferative activity associated with the accumulation of additional genetic lesions [16].

In 2005 Baxter et al. showed JAK2 mutation in 97% of PV, 57% of ET, and 50% of PMF cases [17]. Three other groups showed high frequency of JAK2 mutation in MPNs in the same year [18,19,20]. We found JAK2 mutation in 86.2% of the PV group, 58.5% of the ET group, and 70.6% of PMF group. The frequency of JAK2 mutation in the PMF group was higher than that reported in the literature. All these patients with PMF were JAK2 heterozygous mutant. Cytogenetic analysis of the bone marrow was performed for 61 patients. Among these patients, 8 (13%) of them had genetic abnormalities. The number of patients with genetic abnormalities was not high enough to make prognostic assumptions about these abnormalities.

Thromboembolic complications were seen in 36.7% of all MPN patients in our study. In the literature, thromboembolic complications at diagnosis range from 7% to 57% and rise to 41%-91% during follow-up [13,21,22,23,24]. In our study, thromboembolic complications were more frequent in ET cases, but the difference did not reach statistical significance. Both in PV and ET arterial thrombotic events were much more frequent than venous ones. This finding was consistent with prospective studies [25,26]. In patients with ET, arterial events were mostly located in cerebral arteries. In the study of Pósfai et al., 18 out of 102 ET patients had stroke. They found that stroke was not related to platelet number but rather to JAK2 mutation status [27]. Both the frequency of stroke in ET and the risk factors were similar to our results. In patients with PV, cardiac events were more frequent than in patients with ET or PMF. Ten patients had both arterial and venous vascular events, and 7 of these patients had ET. Incidence of thrombotic events in PMF patients was as high as in other MPN groups. There was no significant difference between venous and arterial thrombotic events in PMF patients. Barbui et al. published a study in 2010 reporting fewer thromboembolic events in PMF compared to other MPNs [28]. In our study, we included thromboembolic events at both diagnosis and follow-up, and this might have affected the results.

After univariate analysis, older age, higher leukocyte count at diagnosis, and JAK2 positivity were risk factors for thrombosis. Increasing age is shown to be a risk factor in large cohorts of patients with MPNs [25,29]. In the ECLAP study, risk increment was shown in PV patients above the age of 65, and in the IPSET-Thrombosis study age above 60 was shown to be an independent risk factor in ET patients [25,29]. In our study, the polycythemia vera199 age of patients with thrombosis was 63.5 years. Leukocytes and especially neutrophils play a major role in inflammatory response and activation of the coagulation system [30]. Barbui et al. found leukocytosis as an independent risk factor for arterial thrombosis in MPN [31]. Increased baseline leukocyte count was shown be a risk factor for thrombosis in ET patients in other large cohort studies [32,33,34]. In our study the median baseline leukocyte count was 13.8x109/L in patients with thrombosis. Leukocyte count higher than 15x109/L was shown to be a risk factor for cardiac events in PV patients [35]. We did not find any significant relationship between platelet and hemoglobin levels and thrombotic events. Cytotoxic therapy is recommended in patients who are at a high risk of thrombosis [9]. Based on our findings we think, like many other authors, that leukocytosis is a novel high risk factor for thrombosis and cytotoxic therapy should be started in patents with persistent leukocytosis, and during follow-up, leukocyte count target is as important as hemoglobin and platelet targets.

JAK2 positivity was shown to increase both arterial and venous thrombosis by 2-fold in ET patients [36]. Barbui et al. showed the risk increment in PMF patients with JAK2 positivity and showed that there is an even higher incidence of thrombotic events if the mutation is together with leukocytosis [28]. In our study, 36.7% of the patients who were heterozygous mutant for JAK2 had a thrombotic event. On the other hand, only 16.7% of JAK2 mutation-negative patients had thrombosis (p=0.041). Allele burden was not different between cases with and without thrombosis, which is controversial in some studies [37]. De Grandis et al. recently showed that there is an abnormal adhesion of red blood cells to the subendothelial protein laminin via the JAK2V617F pathway in PV patients [38]. There are other studies showing that platelet and leukocyte functions and plasma hypercoagulation markers are altered by JAK2 mutation in a prethrombotic way [39,40]. Today, JAK2 mutation monitoring during follow-up is not recommended [9]. Based on our results and knowledge of the literature, JAK2 mutation positivity could be another high-risk factor for thrombosis along with age, previous thrombosis, and leukocytosis. However, regarding the high frequency of positive results, we cannot recommend cytotoxic treatment for every JAK2 mutation-positive patient.

A total of 101 (34.4%) patients had hemorrhagic episodes. Almost all episodes were mucocutaneous or gastrointestinal. This finding is consistent with the literature [13,22]. The only risk factor for hemorrhage was platelet count over 1000x109/L. Elliot and Tefferi reached the same result [41]. It is thought that during extreme thrombocytosis the reduced levels of high-molecular-weight von Willebrand (vW) factor causes acquired vW disease, which is responsible for bleeding tendency. Frequency of hemorrhagic and thrombotic episodes was virtually the same among the MPN groups.

Hydroxyurea was the most commonly used agent in all MPN groups. Anagrelide was almost always used in ET cases. Patients with ET who were using anagrelide were significantly younger than the other ET patients. Our treatment choices correlated nicely with the current recommendations. Hydroxyurea is the initial choice of treatment because of its proven efficiency, especially in reducing thrombotic risk [42,43]. However, hydroxyurea is recommended to be used with caution in young patients regarding the data showing a risk increment of leukemia in long-term usage of hydroxyurea by Kiladjian et al. [8]. In the ANAHYDRET study, it was shown that anagrelide is as effective as hydroxyurea [44]. Secondary leukemia has not been reported with anagrelide treatment yet. Interferon alpha was the least commonly used agent in all MPN groups, probably because of its parenteral usage and poor tolerability. In our study, we did not find any relation between the complications of MPN and the treatment methods.

Survival was longest in PV and shortest in PMF cases. Polycythemia vera has a life expectancy of 10 to 20 years according to the literature and our finding was consistent with this knowledge [45]. Although we found that patients with PMF had the shortest survival, their mean overall survival was 11.4 years, which is longer than 5.5 years as reported in the literature [46]. None of the PMF cases transformed to leukemia and this could be one of the reasons for the finding above. Life expectancy of ET patients ranges from 13 to 22.3 years according to the literature [14,47,48]. In our ET patient group, mean overall survival was 14.9 years. Although the survival of ET patients was shorter than that of PV patients, this was not statistically significant.

Older age, leukocytosis, and low hemoglobin and high platelet count were found to be related to survival in ET patients in the literature [48,49]. In our study, patients older than 60 years at diagnosis had shorter survival than those younger than 60. We also found that anemia (defined as hemoglobin of <13 g/dL) was related to shorter survival.

Leukemic transformation shortened survival significantly, as expected. However, there were only 7 patients who developed leukemia. Therefore, risk factors for leukemic transformation and their effects on survival could not be examined properly.

Patients with thromboembolic events had shorter overall survival. This effect of thromboembolism was most significantly seen in the ET subgroup. This finding is consistent with other reports [48,50]. ET is a disease that has a life expectancy of 20 years, as mentioned before, and thromboembolism is a preventable complication of ET, so therapy should be directed toward preventing thromboembolism in ET.

In conclusion, thromboembolic complications are the most important preventable risk factors for morbidity and mortality in MPNs. Leukocytosis and JAK2 positivity are risk factors for thrombosis and may be more important than the elevated hemoglobin levels and platelet counts.

## Ethics

Ethics Committee Approval: Study was approved by Ethics Committees of Eskişehir Osmangazi University and Aydın Adnan Menderes University; Informed Consent: Informed consent was not applicable.

## AUTHORSHIP CONTRIBUTIONS

Medical Practices: Mustafa Ünübol, Eren Yağcı, Neslihan Andıç; Concept: Neslihan Andıç, Mustafa Ünübol, Eren Yağcı, Olga Meltem Akay; Design: Neslihan Andıç, Mustafa Ünübol, Eren Yağcı, Olga Meltem Akay; Data Collection or Processing: Neslihan Andıç, Mustafa Ünübol, Eren Yağcı, Olga Meltem Akay, İrfan Yavaşoğlu, Vefki Gürhan Kadıköylü, Ali Zahit Bolaman; Analysis or Interpretation: Neslihan Andıç, Vefki Gürkan Kadıköylü; Literature Search: Neslihan Andıç, Mustafa Ünübol, Eren Yağcı, Olga Meltem Akay, İrfan Yavaşoğlu, Vefki Gürhan Kadıköylü, Ali Zahit Bolaman; Writing: Neslihan Andıç, Mustafa Ünübol, Eren Yağcı, Olga Meltem Akay, İrfan Yavaşoğlu, Vefki Gürhan Kadıköylü, Ali Zahit Bolaman.

Conflict of Interest: The authors of this paper have no conflicts of interest, including specific financial interests, relationships, and/or affiliations relevant to the subject matter or materials included.

## Figures and Tables

**Table 1:**
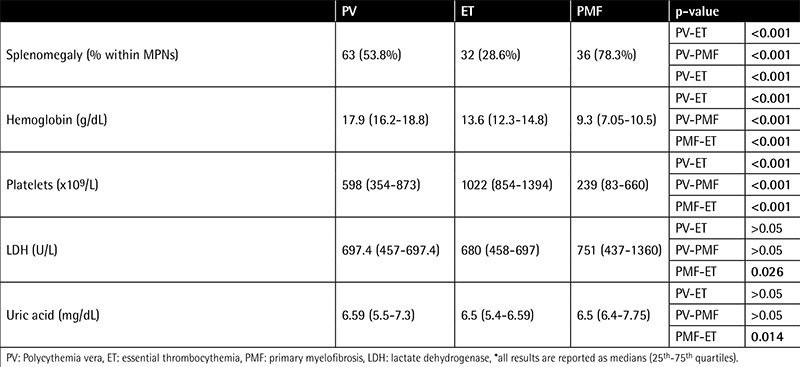
Clinical and laboratory characteristics of patients with myeloproliferative neoplasm.*

**Table 2:**
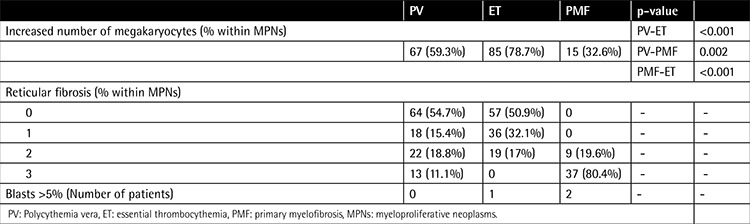
Bone marrow findings at diagnosis.

**Table 3:**
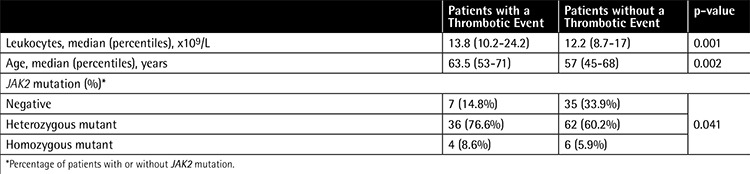
Risk factors for thrombosis.

**Table 4:**
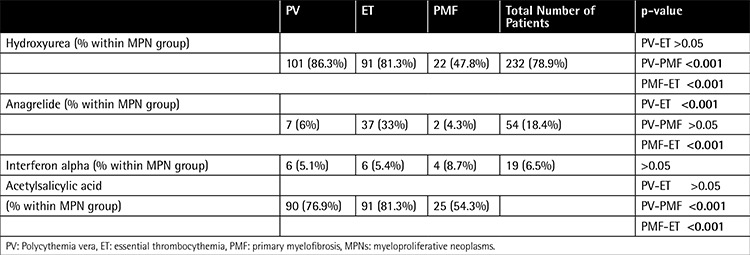
Treatment modalities in myeloproliferative neoplasms.

**Table 5:**
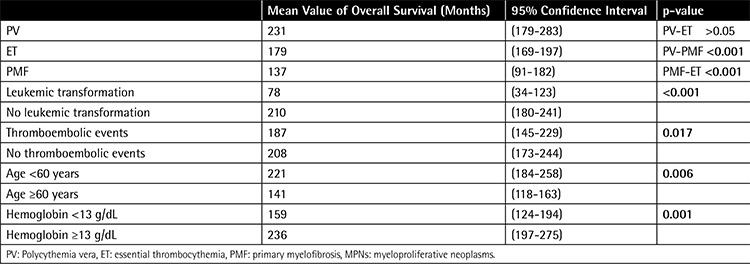
Factors affecting survival in myeloproliferative neoplasm cases.
